# Squarate-Based Isoreticular Analogue of CALF-20: Fact or Fiction?

**DOI:** 10.1021/acs.cgd.6c00193

**Published:** 2026-08-06

**Authors:** Francesco Della Croce, Giulio Bresciani, Marco Taddei

**Affiliations:** † Department of Chemistry and Industrial Chemistry, INSTM Research Unit, 9310 University of Pisa, Via G. Moruzzi 13, 56124 Pisa, Italy; ‡ Centro per l’Integrazione della Strumentazione scientifica dell’Università di Pisa (C.I.S.U.P.), University of Pisa, 56124 Pisa, Italy

## Abstract

The hypothetical squarate-based isoreticular analogue of benchmark metal–organic framework (MOF) CALF-20 was recently predicted by computations to outperform the parent material as a sorbent for CO_2_ capture. In this work, we performed a synthetic screening aimed at synthesizing this hypothetical MOF, which failed to afford the desired product and led to the discovery of two new nonporous coordination polymers, named **UdP-40** and **UdP-41**, with the same 2:2:1 Zn/1,2,4-triazolate/squarate stoichiometry expected for a CALF-20 analogue, but different crystal structure. **UdP-40** and **UdP-41** were characterized by elemental analysis, thermogravimetric analysis, variable temperature powder X-ray diffraction,infrared spectroscopy and CO_2_ sorption analysis, and their structures were solved from powder and single crystal X-ray diffraction, respectively. Both compounds crystallize in orthorhombic space groups and display the same peculiar tetradentate coordination mode for squarate, involving two monodentate O atoms and one bridging O atom, while leaving one free O atom, leading to the formation of previously unreported topologies. **UdP-40** features one coordinated water molecule per formula unit, whose removal at temperatures above 250 °C leads to complete amorphization.

## Graphical abstract

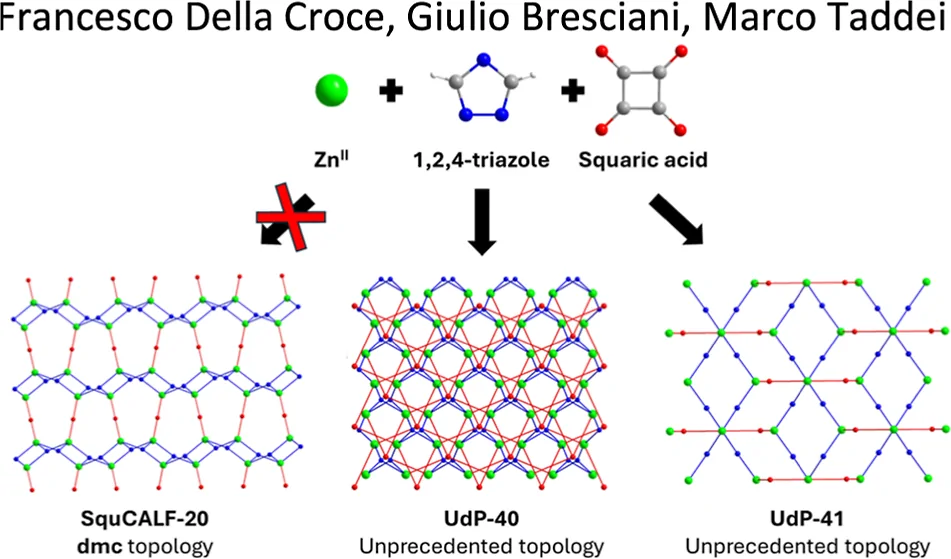

## Introduction

Adsorption-based postcombustion CO_2_ capture is seen as a potential alternative to classical amine scrubbing, employing solid sorbents such as zeolites, porous carbons, amine-modified silicas and metal–organic frameworks (MOFs).^1–4^ The typical CO_2_ concentration range in flue gas is 4–25% and physisorbents, i.e., solids that adsorb CO_2_ through van der Waals interactions with their surface, are usually preferred for their relatively low heat of adsorption, which allows less energy intensive regeneration. Zeolite 13X has traditionally been the workhorse in adsorption-based CO_2_ capture, because of its favorable interaction with CO_2_, high CO_2_/N_2_ selectivity and low cost.^5–7^ However, its strong hydrophilicity makes it ineffective when the gas stream is humid, with water outcompeting CO_2_ for the same adsorption sites.^8,9^

MOFs have been thoroughly investigated as alternative sorbents to zeolites, but water has remained the Achilles’ heel, either because it can degrade the MOF, or because it poisons the adsorption sites, similar to the case of zeolite 13X.^10^ The recent discovery of CALF-20, a MOF based on Zn^II^, 1,2,4-triazolate (Htrz) and oxalate of formula Zn_2_(trz)_2_(C_2_O_4_), has marked a turning point, as it combines a series of highly desirable features: it can uptake large amounts of CO_2_ at partial pressures compatible with real applications, it has a high CO_2_/N_2_ selectivity, it maintains its performance until 44% relative humidity, and it can be conveniently produced at a low cost and in large amounts.^11,12^ CALF-20 displays a crystal structure where Zn-triazolate layers are pillared by chelating oxalate ligands, giving rise to a 3D network of interconnected channel-like ultramicropores with 3,4-connected **dmc** topology (Figure S1).

Since the first report of CALF-20, several works have appeared in the literature, focusing on understanding the structural response to CO_2_ and H_2_O adsorption,^13–16^ identifying the best process parameters to decrease the cost of CO_2_ capture^17^ and tuning the physicochemical properties by changing the triazolate linker.^18,19^ A recent computational work^20^ proposed the replacement of oxalate with both a range of other dicarboxylates and squarate, predicting the adsorption properties using Grand-Canonical Monte Carlo and suggesting that the hypothetical squarate-based CALF-20 (SquCALF-20) could outperform the parent material in terms of CO_2_ uptake (almost 5 mmol g^–1^ at 1 bar and 293 K) and CO_2_/N_2_ selectivity (exceeding 400). SquCALF-20 was predicted to be synthetically accessible based on a similar computed formation energy to CALF-20. A subsequent computational work^21^ examined the same series of sorbents for CO_2_/CH_4_ separation, predicting a much lower CO_2_ uptake for SquCALF-20 (about 2.8 mmol g^–1^ at 1 bar and 298 K) and a moderate CO_2_/CH_4_ selectivity (about 9). Another computational work^22^ looked at the effect of the linker backbone on the CO_2_ adsorption properties, exploring diazenedicarboxylate, 1,2-hydrazinedicarboxylate, acetylenedicarboxylate and fumarate. SquCALF-20 was included as a term of comparison alongside the parent CALF-20, based on the exceptional performance predicted in ref 20.

While several Zn-trz-dicarboxylate MOFs were previously synthesized, with the same **dmc** topology of CALF-20,^23–27^ to the best of our knowledge the only instance where squarate (sqa^2–^) was experimentally employed as the oxygenated ligand appeared very recently in a paper by Tian et al.^28^ The authors used 3-amino-1,2,4-triazole (Hatrz) and obtained a porous framework of formula Zn_2_(atrz)_2_(sqa) (named MAF-60) where the sqa linker acts as a 3-connected node and two crystallographically independent Zn atoms are present, one of which is 4-connected and the other is 5-connected, thus giving rise to a 3̂^2^,4,5-connected topology, different from that of CALF-20. MAF-60 was investigated for propylene/propane separation, displaying exceptional selectivity toward propylene.

Motivated by the interest aroused by the hypothetical MOF SquCALF-20, we set out to investigate its synthetic feasibility and, in case of success, to assess whether its gas adsorption properties align with the computational predictions.

## Experimental Section

### Materials and Methods

All reagents and solvents were purchased from Alfa Aesar, Merck, Strem, FluoroChem, or TCI Chemicals and were used as received without further purification.

Powder X-ray diffraction (PXRD) patterns were recorded over the 3–40° 2θ range on a Rigaku MiniFlex 600C diffractometer using Cu Kα radiation (λ = 1.54056 Å). The X-ray tube operated at 40 kV and 15 mA. Variable temperature PXRD patterns were recorded using an Anton Paar BTS500 camera equipped with a ceramic sample holder. The PXRD pattern of UdP-40 used for structure solution and Rietveld refinement was collected in the 10–120° 2θ range, with a 0.02° step size and 1° min^–1^ scan rate.

Carbon, hydrogen and nitrogen (CHN) analysis were performed on a Vario MICRO cube instrument (Elementar).

Attenuated total reflectance infrared (ATR–IR) spectra were collected on an Agilent Cary 630 spectrometer equipped with a diamond crystal in the 700–4000 cm^–1^ wavenumber range with a resolution of 1 cm^–1^.

Thermogravimetric analysis (TGA): TGA was performed in air with a TA Instrument Thermo balance model Q5000IR using 10–15 mg of sample, with a heating rate of 10 °C min^–1^ up to 700 °C.

CO_2_ isotherms were collected at 0 °C. To keep isothermal conditions, the sample was inserted into a chiller dewar from Micromeritics in which a coolant or heating fluid, connected to a thermostatic bath (JULABO F25), can recirculate. The samples (70-100 mg) were activated at 120 °C under dynamic vacuum for 6 h prior to the analysis.

### Synthesis of Zn­(H_2_O)_2_(sqa)

Zn­(NO_3_)_2_·6H_2_O (595 mg, 2.0 mmol) was dissolved in H_2_O (1.0 mL) inside a glass vial and heated to 90 °C. H_2_sqa (114 mg, 1.0 mmol) was dissolved in H_2_O (4.0 mL) inside a glass vial, mixed with a solution of Htrz (138 mg, 2.0 mmol) in H_2_O (1.0 mL) and heated to 90 °C. The two mixtures were combined and left to react for 3 h at 90 °C under stirring. The white solid was recovered by centrifugation, washed with water and dried in an oven at 80 °C. Yield: 88 mg (41%, based on H_2_sqa).

### Synthesis of Zn_2_(trz)_2_(sqa)­(H_2_O) (**UdP-40**)

Zn­(H_2_O)_2_(sqa) (100 mg, 0.47 mmol) was suspended in 5 mL of a 1 M solution of Htrz (5.0 mmol) for 3 h at 90 °C under stirring. The white solid was recovered by centrifugation, washed with water and acetone and dried in an oven at 60 °C. Yield: 80 mg (41%, based on sqa^2–^).

Elemental Analysis: Found: C, 22.44; H, 1.68; N, 18.33; Calcd for Zn_2_(trz)_2_(C_4_O_4_)­(H_2_O)_2_: C, 23.13; H, 1.93; N, 20.24.

### Synthesis of Zn_2_(trz)_2_(sqa) Single Crystals (**UdP-41**)

ZnCO_3_ (251 mg, 2.0 mmol), H_2_sqa (114 mg, 1.0 mmol) and Htrz (138 mg, 2.0 mmol) were suspended in H_2_O (14 mL) inside a Teflon-lined stainless steel hydrothermal bomb and heated to 200 °C for 24 h. The colorless block crystals were recovered by filtration, washed with water and dried in an oven at 80 °C. Yield: 275 mg (73%).

Elemental Analysis: Found: C, 25.20; H, 0.93; N, 20.65; Calcd for Zn_2_(trz)_2_(C_4_O_4_): C, 25.33; H, 1.06; N, 22.16.

### Structure Solution and Refinement of **UdP-40**

The PXRD pattern of **UdP-40** was indexed using TREOR,^29^ finding an orthorhombic unit cell with lattice parameters *a* = 11.0911 Å, *b* = 21.7907 Å, *c* = 10.7541 Å and a figure of merit F20 of 33. Analysis of the systematic extinctions suggested *Fdd*2 as the most probable space group. The crystal structure was solved ab initio with a real-space global optimization approach, using the Parallel Tempering algorithm^30^ implemented within FOX.^31^ The best structural model was obtained by employing one fragment consisting of the trz linker with one Zn atom bonded to one of the N atoms and one sqa linker with center of mass located on Wickoff position 8b, with coordinates (0, 0, *z*), lying on a 2-fold axis. A Ne atom with occupancy 0.8 was used to represent the O atom of a water molecule, which allows to set specific antibump restraints between water and the framework.

The structure was refined using the Rietveld method in the software TOPAS Academic (version 6.0).^32^ The background was refined using 8 parameters, the profile function was refined using 8 parameters, and sample displacement correction was used. The positions and atomic displacement parameters of the Zn and Ow (coordinated water) atoms were left free to refine.

To correctly describe the geometry of the trz linker, a rigid group was employed. One dummy atom, named XX, whose occupancy factor was set to zero, was employed in the rigid group and placed at the center of the ring. Translational and rotational parameters of the rigid group were left free to refine. The atomic displacement parameters of all atoms of the trz linker were constrained to have the same value.

The following z matrix was used to define the rigid group:

z_matrix XX

z_matrix N3 XX 1.12814

z_matrix N4 N3 1.36000 XX 51

z_matrix C2 N3 1.30000 N4 105 XX 0

z_matrix N1 C2 1.33000 N3 114 N4 0

z_matrix C5 N1 1.33000 C2 103 N3 0

To correctly describe the geometry of the sqa linker, a rigid group was employed. One dummy atom, named YY, whose occupancy factor was set to zero, was employed in the rigid group and placed at the center of the ring. The translational parameters along the *x* and *y* axis direction were constrained to have the same value, while the parameter along the *z* axis direction was left free to refine. The rotational parameters along the *x* and *y* axis direction were set to zero, whereas the parameter along the *z* axis direction was left free to refine. The atomic displacement parameters of all atoms of the sqa linker were constrained to have the same value.

The following z matrix was used to define the rigid group:

z_matrix YY

z_matrix C6 YY 1.05

z_matrix C7 C6 1.45 YY 45

z_matrix O10 C7 1.24 C6 135 YY 180

z_matrix C8 C7 1.45 C6 90 YY 0

z_matrix O11 C8 1.24 C7 135 O10 0

z_matrix O9 C6 1.24 C7 135 O10 0

At the end of the refinement, all the parameters were refined together until convergence.

### Structure Solution and Refinement of **UdP-41**

Single-crystal X-ray diffraction (SCXRD) data were collected on a Bruker D8 Venture diffractometer equipped with a Mo Kα microfocus source (λ = 0.71073 Å) and a Photon II 2D detector. Crystals were mounted on MiTeGen loops of appropriate size and coated with Cargille Immersion Oil (type NVH-658). Unit-cell determination and initial refinement were carried out using APEX4.^33^ Data integration and reduction were performed with SAINT^34^ and XPREP,^35^ and absorption corrections were applied using SADABS.^36^ Structures were solved via intrinsic phasing with ShelXT^37^ and refined by full-matrix least-squares methods. All non-hydrogen atoms were refined anisotropically. Hydrogen atoms were placed in calculated positions and refined using a riding model.

Crystallographic data for **UdP-40** and **UdP-41** have been deposited at the Cambridge Crystallographic Data Centre under the deposition numbers 2504491 and 2504428, respectively, and can be obtained free of charge via http://www.ccdc.cam.ac.uk. Tables 1 and 2 report crystallographic details for **UdP-40** and **UdP-41**, respectively. The Rietveld plot for **UdP-40** is displayed in Figure S2.

**Table 1:** Crystallographic Parameters and Refinement Details of **UdP-40**

compound	**UdP-40**
CCDC deposition no.	2504491
formula	C_8_H_8_N_6_O_6_Zn_2_
formula weight, g mol^–1^	414.96
wavelength, Å	1.54056
crystal system	orthorhombic
space group	*Fdd*2
*a*, Å	11.0998(3)
*b*, Å	21.8126(5)
*c*, Å	10.7681(3)
cell volume, Å^3^	2607.1(1)
*Z*	8
density, g cm^–3^	2.113
*R*_p_	0.07746
*R*_wp_	0.10478
*R*_Bragg_	0.03681
GOF	2.600

**Table 2:** Sample, Crystal Data, Data Collection and Structure Refinement for **UdP-41**

compound	**UdP-41**
CCDC ID	2504428
formula	C_8_H_4_N_6_O_4_Zn_2_
FW, g mol^–1^	378.91
*T*, K	298(2)
λ, Å	0.71073
crystal system	orthorhombic
space group	*Imma*
*a*, Å	9.6048(2)
*b*, Å	7.2600(2)
*c*, Å	16.8190(4)
cell volume, Å^3^	1172.80(5)
*Z*	4
density, g cm^–3^	2.146
absorption coefficient, mm^–1^	4.114
*F*(000)	744
crystal size, mm	0.049 × 0.041 × 0.040
θ limits, deg	2.42 to 28.68
reflections collected	9808
independent reflections	836 [*R*(int) = 0.0386]
data/restraints/parameters	836/0/64
Goodness of fit on *F* ^2^	1.164
*R*_1_ (*I* > 2σ(*I*))	0.0203
w*R*_2_ (*I* > 2σ(*I*))	0.0519
*R*_1_ (all data)	0.0208
w*R*_2_ (all data)	0.0522
largest diff. peak and hole, e Å^–3^	0.367 and −0.486

## Results and Discussion

### Synthetic Screening

We started our investigation by replacing oxalic acid with H_2_sqa in synthetic conditions similar to those reported for CALF-20 in a 2019 patent,^38^ that is, reacting ZnCO_3_, Htrz and H_2_sqa in a 2:2:1 ratio in 3.0 mL of a mixed water/ethanol (1:2 V/V) solvent at 90 °C for 1 h in a glass vial, followed by 2 h at RT under stirring (Table 3, entry 1). We obtained a white solid whose powder X-ray diffraction (PXRD) pattern cannot be fitted using the lattice parameters obtained by Gopalsamy et al.^20^ by DFT optimization of the structure of SquCALF-20 (monoclinic, *P*2_1_/*c*; *a* = 10.502 Å, *b* = 10.301 Å, *c* = 9.446 Å, β = 124.6°, Figure S3). In addition, the presence of two different crystalline phases was apparent. The reaction was repeated using only water as the solvent and increasing the total volume to 6.0 mL to facilitate the dissolution of H_2_sqa (Table 3, entry 2). Upon stirring at 90 °C for 3 h, the white solid recovered displayed a PXRD pattern where only one of the two phases previously observed was present (Figure S4). The crystallinity of this new phase, named **UdP-40** (where UdP stands for Università di Pisa), was not sufficient to allow indexing of the PXRD pattern, and reflections possibly belonging to a minor secondary phase were also present. Performing the synthesis in aqueous medium at RT for 24 h also afforded **UdP-40** (Table 3, entry 3), still displaying spurious reflections (Figure S4).

**Table 3:** Screening of Reaction Conditions^e^

entry	Zn precursor	solvent	NaOH (mmol)	*T* (°C)	time (h)	product
1^a^	ZnCO_3_	H_2_O/EtOH (1:2)	/	90 + RT	1 + 2	**UdP-40 + unknown phase**
2	ZnCO_3_	H_2_O	/	90	3	**UdP-40** ^b^
3	ZnCO_3_	H_2_O	/	RT	24	**UdP-40** ^b^
4	ZnCO_3_	H_2_O	/	200	24	**UdP-41**
5	Zn(NO_3_)_2_·6H_2_O	H_2_O	/	90	3	**Zn(H**_2_ **O)**_2_ **(sqa)**
6	Zn(NO_3_)_2_·6H_2_O	H_2_O	1	90	3	**Zn(H**_2_ **O)**_2_ **(sqa)**
7	Zn(NO_3_)_2_·6H_2_O	H_2_O	2	90	3	**UdP-40** ^b^
8^c^	Zn(H_2_O)_2_(sqa)	H_2_O	/	90	3	**UdP-40**
9	Zn(NO_3_)_2_·6H_2_O	DMF	/	90	3	**UdP-41** ^b^
10	ZnCO_3_	DMF	/	90	3	**UdP-41**
11^d^	Zn(NO_3_)_2_·6H_2_O	DMF/MeOH (1:1)	/	120	18	**UdP-41**

a *V* = 3.0 mL.

b Traces of a minor spurious phase.

c *n*(Zn^II^) = 0.5 mmol, *n*(Htrz) = 5 mmol, *V* = 5 mL.

d *n*(Zn^II^) = 0.3 mmol, *n*(Htrz) = 1.5 mmol, *n*(H_2_sqa) = 0.3 mmol, *V* = 6.0 mL.

e Unless otherwise specified, the reaction mixture is composed as follows: *n*(Zn^II^) = 2.0 mmol, *n*(Htrz) = 2.0 mmol, *n*(H_2_sqa) = 1.0 mmol, *V* = 6.0 mL.

With the aim of obtaining a phase-pure and highly crystalline **UdP-40**, we increased the reaction temperature to 200 °C, leading to the formation of single crystals (Table 3, entry 4), whose PXRD pattern was different from both SquCALF-20 and **UdP-40** (Figure 1). The structure of this new phase, named **UdP-41**, was solved from single crystal XRD data (vide infra).

**Figure 1:**
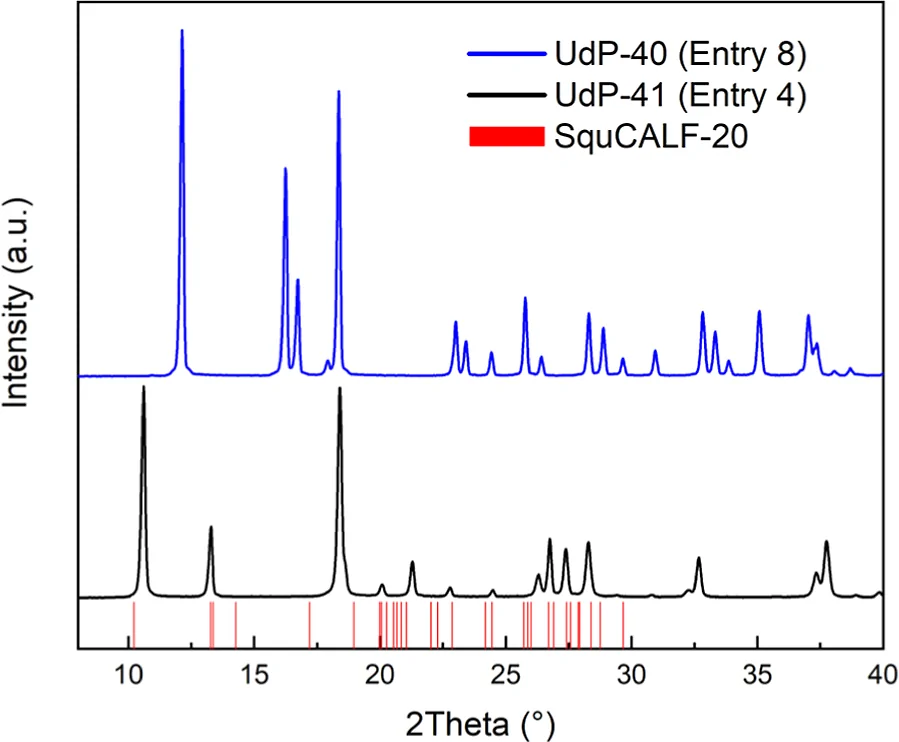
Comparison between experimental PXRD patterns of **UdP-40** (blue), **UdP-41** (black) and calculated reflections for SquCALF-20 (red) reported in ref 20.

Further experiments were carried out at 90 °C starting from an acidic and water-soluble Zn precursor, i.e., Zn­(NO_3_)_2_·6H_2_O, obtaining a solid with a PXRD consistent with the Zn-squarate of formula Zn­(H_2_O)_2_(sqa) and having a sodalite-like topology, previously reported by Cheetham and co-workers and obtained in much harsher conditions^39^ (Figure S5 and Table 3, entry 5). Given that working with Zn­(NO_3_)_2_·6H_2_O did not lead to the inclusion of trz, reactions were performed adding an increasing amount of NaOH to the reaction mixture, i.e., 0.5 and 1 mol equiv (eq) with respect to Zn. The synthesis with 0.5 equiv of NaOH still afforded phase-pure Zn­(H_2_O)_2_(sqa), whereas nearly phase-pure **UdP-40** was obtained in the presence of 1 equiv of NaOH (Figure S5 and Table 3, entries 6 and 7).

Next, we sought to find out whether it is possible to recrystallize phase-pure **UdP-40** by treatment of Zn­(H_2_O)_2_(sqa) with Htrz. To our delight, suspending Zn­(H_2_O)_2_(sqa) in a 1 M aqueous solution of Htrz at 90 °C for 3 h afforded a phase-pure **UdP-40** whose high crystallinity enabled the indexing of the PXRD pattern and subsequent structure solution (vide infra) (Figure 1 and Table 3, entry 8).

The effect of the solvent was explored by reacting either Zn­(NO_3_)_2_·6H_2_O or ZnCO_3_ with Htrz and H_2_sqa in DMF, leading to obtain **UdP-41**, regardless of the Zn^II^ precursor employed (Figure S6 and Table 3, entries 9 and 10). We also tested whether the reaction conditions that lead to the formation of MAF-60 could be suitable for the attainment of an analogue containing trz in place of atrz, still obtaining **UdP-41** (Figure S6 and Table 3, entry 11).

### Characterization

CHN elemental analysis suggested that **UdP-40** and **UdP-41** have the same 2:2:1 Zn/trz/sqa ratio found in CALF-20, with **UdP-40** containing one water molecule per Zn, whereas **UdP-41** does not contain water. According to thermogravimetric analysis (TGA) (Figure 2a), **UdP-40** loses 9.2% of its initial mass at 250 °C, corresponding to one water molecule per Zn [calculated loss for the formula Zn_2_(trz)_2_(sqa)­(H_2_O): 8.7%], while the organic part of the framework starts degrading above 400 °C, leaving behind 38.1% of the initial mass in the form of ZnO (calculated residual mass of ZnO: 39.2%). The release of water at such a high temperature suggests that it is coordinated to Zn. In contrast, **UdP-41** displays a single mass loss above 400 °C, when the organic part of the framework is degraded, leading to the formation of a residual of ZnO accounting for 45.3% of the initial mass, in good agreement with the calculated one (42.9%). Variable temperature PXRD performed in the 40–280 °C range (Figure 2b,c) reveals that **UdP-40** is stable until 240 °C and becomes completely amorphous at 280 °C, suggesting that the release of coordinated water is accompanied by structural collapse, whereas **UdP-41** undergoes no changes until 400 °C. ATR-IR analysis confirms the presence of water in **UdP-40** (Figure 2d), revealed by the broad absorption band in the 3500–3000 cm^–1^ range, attributed to the O–H stretching vibration, and the band at 750 cm^–1^, attributed to the rocking deformation of coordinated water.^40,41^ Many bands are present in both spectra, however, **UdP-41** displays a band at 1640 cm^–1^ that is not present in **UdP-40**. We assign this band to the stretching vibration of noncoordinating C=O groups of sqa ligands. These groups are hydrogen-bonded with water in **UdP-41**, leading to a decrease of their stretching frequency that most likely shifts the relative band to the region where the coordinated C=O groups resonate (see discussion herein for further structural details). CO_2_ adsorption isotherms collected at 0 °C up to 1 bar display no appreciable uptake for either UdP-40 or UdP-41, whereas CALF-20 adsorbs up to 5.1 mmol g^–1^ in the same conditions (Figure 2e).^42^

**Figure 2:**
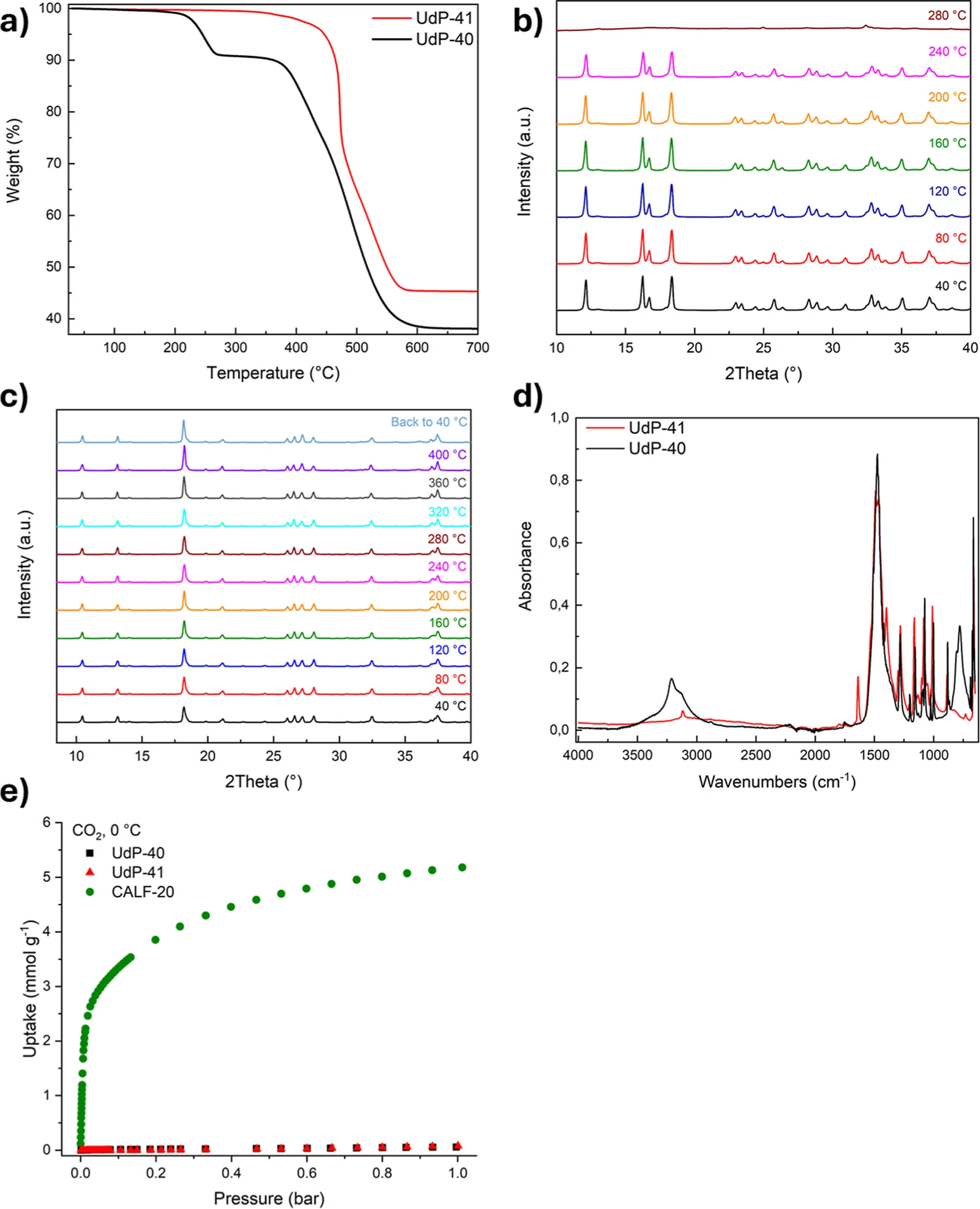
TGA curves of **UdP-40** (black) and **UdP-41** (red) (a), VT-PXRD of **UdP-40** (b), VT-PXRD of **UdP-41** (c), IR spectra of **UdP-40** (black) and **UdP-41** (red) (d), CO_2_ adsorption isotherms of **UdP-40** (black), **UdP-41** (red) and CALF-20 (green) collected at 0 °C (e).

### Crystal Structures

**UdP-40** crystallizes in the face-centered orthorhombic space group *Fdd*2 (number 43), with the following lattice parameters: *a* = 11.0998(3) Å, *b* = 21.8126(5) Å, *c* = 10.7681(3) Å, *V* = 2607.1(1) Å^3^. The asymmetric unit is composed of one Zn atom, one trz molecule, one coordinated water molecule and half of a sqa molecule (Figure 3a). Atoms C6, C8, O9 and O11 of sqa occupy Wickoff position 8b, with coordinates (0, 0, *z*) lying on a 2-fold axis. Zn is in a distorted octahedral coordination geometry, with three N atoms from trz coordinated in a *mer* fashion (Zn–N distances of 2.045 Å, 2.078 Å and 2.195 Å, respectively), one terminal water molecule (Zn–O distance of 2.187 Å) and two O atoms from sqa (Zn–O distances of 2.188 Å and 2.379 Å, respectively) (Figure 3b). Each trz ligand binds three different Zn atoms (Figure S7), whereas the sqa ligand displays a peculiar tetradentate binding mode, coordinating to two Zn atoms via crystallographically equivalent monodentate O10 atoms and two Zn atoms via bridging O11 atoms, and leaving its fourth O atom (O9) uncoordinated (Figure S7). The coordinated water molecule is hydrogen bonded to one uncoordinated O9 atom (Ow-O9 distance of 2.696 Å) and to one coordinated O10 atom (Ow-O10 distance of 2.752 Å) from two distinct sqa ligands (Figure S7). Each uncoordinated O9 atom is involved in two hydrogen bonds with two distinct coordinated water molecules (Figure S7). The isolated Zn-trz and Zn-sqa sublattices extend over three and two dimensions, respectively, giving rise to a 3D framework (Figures 3c and S7, S8). The topological reduction of **UdP-40**, performed using the online resource TopCryst,^43^ suggests a previously unreported 3,4,5-connected net (Figure S9).

**Figure 3:**
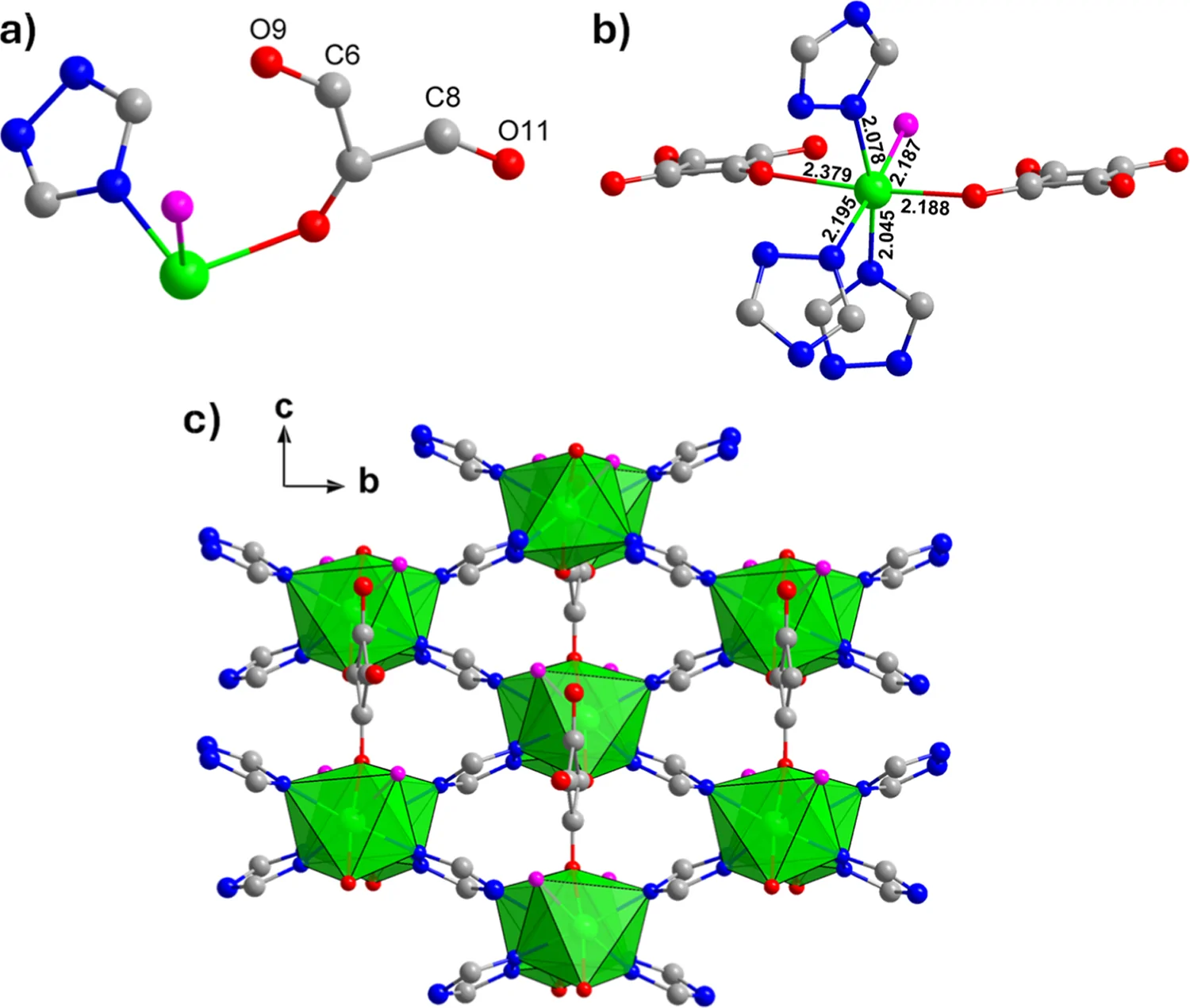
**UdP-40**: asymmetric unit (a), Zn coordination environment (b) and view of the structure along the *a* axis (c). Color code: C, gray; N, blue; O (sqa), red; O (water), pink; Zn, green.

Crystals of **UdP-41** suitable for SCXRD analysis were obtained under the conditions reported in Table 3, entry 4. **UdP-41** crystallizes in the body-centered orthorhombic space group *Imma* (number 74), with the following lattice parameters: *a* = 9.6048(2) Å, *b* = 7.2600(2) Å, *c* = 16.8190(4) Å, *V* = 1172.80(5) Å^3^. The asymmetric unit (Figure 4a) is composed of one Zn atom (Zn1) sitting in a special position lying at the intersection of an inversion center, a 2-fold axis and a mirror plane (Wickoff position 4b, with coordinates 0, 0, 0.5), a second Zn atom (Zn2) sitting in a special position lying over a 2-fold axis and a mirror plane (Wickoff position 4e, with coordinates 0, 0.25, *z*), half of a trz molecule having the N2 atom and the center of mass sitting in special positions lying on a mirror plane (Wickoff position 4b, with coordinates *x*, 0.25, *z*), and half of a sqa molecule sitting on the same mirror plane of Zn2. Zn1 is in a distorted octahedral coordination geometry, with four N atoms from trz coordinated in equatorial positions (Zn–N distance of 2.099 Å) and two O atoms from sqa in axial positions (Zn–O distance of 2.322 Å) (Figure 4b). Zn2 is in a slightly distorted tetrahedral coordination geometry with two N atoms from trz (Zn–N distance of 1.967 Å) and two O atoms from sqa (Zn–O distance of 1.939 Å) (Figure 4b). As usual in these systems, trz is tridentate, binding three different Zn atoms (Figure S10). The sqa ligand displays a very similar tetradentate binding mode to the one seen in **UdP-40** (Figure S10). The isolated Zn-trz and Zn-sqa sublattices extend over two and one dimensions, respectively, giving rise to a 3D framework (Figures 4c and S10, S11). The topological reduction of **UdP-41** suggests a previously unreported 3,4̂^2^,6-connected net (Figure S12).

**Figure 4:**
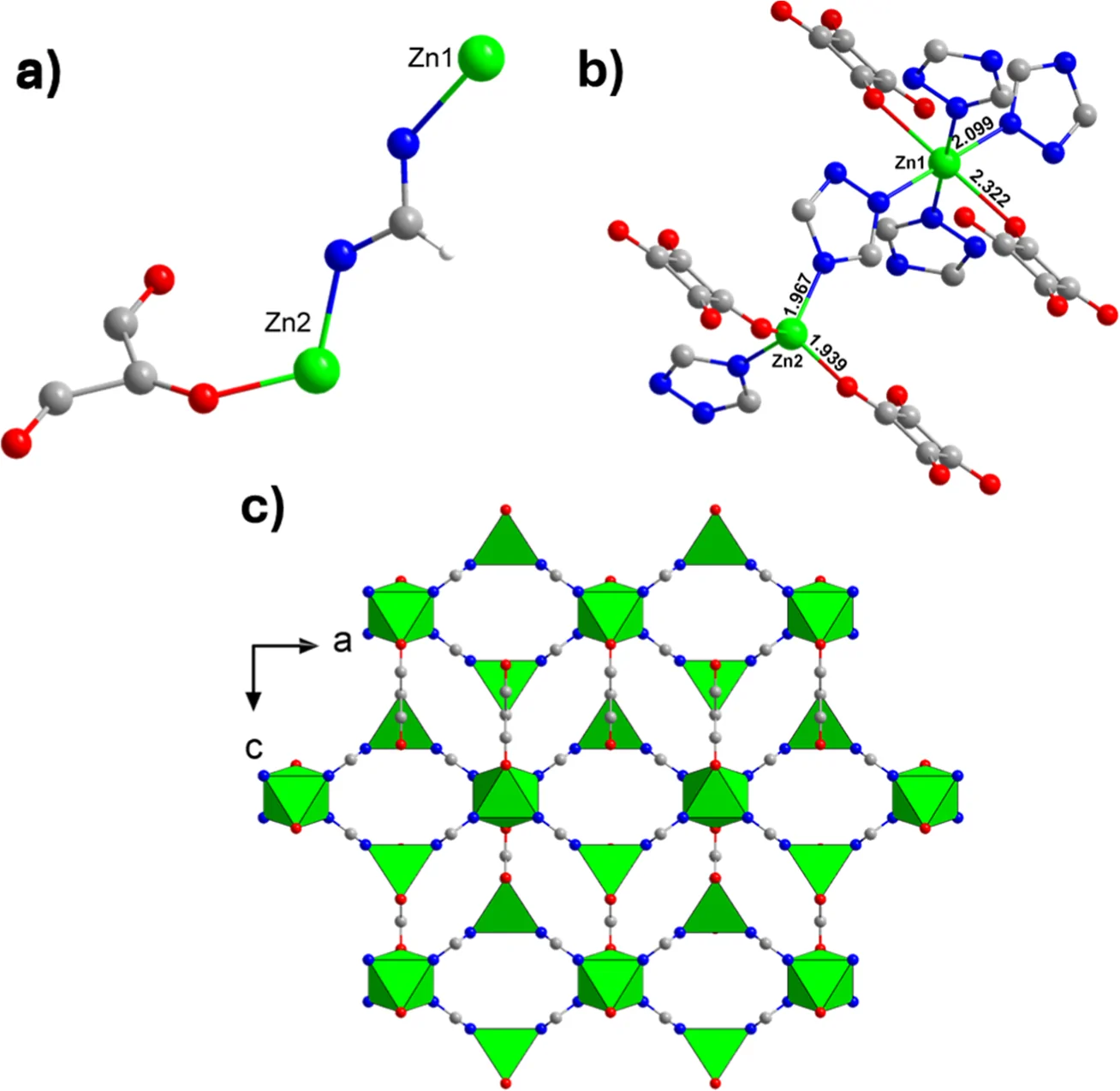
**UdP-41**: asymmetric unit (a), Zn coordination environment (b), and view of the structure along the *b* axis (c). Color code: C, gray; N, blue; O, red; Zn, green; H, white. Hydrogen atoms omitted for clarity in (b,c).

### Discussion

The foregoing results suggest that SquCALF-20 is not synthetically accessible in the parametric space explored here, which is instead dominated by **UdP-40** and **UdP-41**. Water appears to play a key role in driving the self-assembly process: **UdP-40**, containing one coordinated water molecule per formula unit, is only obtained in aqueous environment, whereas DMF and MeOH favor the formation of **UdP-41**. The latter is also obtained under hydrothermal conditions at 200 °C, which have previously been found to prevent the coordination of water to the metal ion in the final crystal structure in other coordination polymers.^44–46^

From the topological standpoint, oxalate acts as a 2-connected node in CALF-20, whereas the sqa linker acts as a 4-connected node in both **UdP-40** and **UdP-41**. A similar coordination mode of sqa is also observed in MAF-60, although the three coordinating oxygen atoms are monodentate in MAF-60, making sqa a 3-connected node (Figure S13). We note that the amino group on the atrz linker likely plays a structure-directing role in MAF-60, as it is involved in a hydrogen bond with the free C=O of the sqa linker (Figure S14).

The different topological function of oxalate and sqa is a consequence of their distinct geometries (Figure S15): oxalate has O–O distances of 2.224–2.665 Å and angles of ca. 120°, making it able to chelate two Zn atoms; sqa has larger average O–O distances of 3.224 Å and angles of ca. 135°, which make it less prone to act as a chelating ligand for Zn. As a matter of fact, no structures exist in the Cambridge Structural Database featuring sqa-Zn chelates.

**UdP-40** and **UdP-41** have densities of 2.113 g cm^–3^ and 2.146 g cm^–3^, respectively, higher than the one predicted for SquCALF-20 (1.381 g cm^–3^)^21^ and that of MAF-60 (1.568 g cm^–3^), and their structures have no accessible porosity, as determined by the software Mercury using a probe with 1.2 Å radius and confirmed by CO_2_ adsorption isotherms (Figure 2e). The removal of coordinated water above 250 °C in **UdP-40** is accompanied by collapse of the framework, as suggested by VT-PXRD analysis (Figure 2b), thus preventing any further assessment of potential porosity on the dehydrated solid.

## Conclusions

In summary, our unsuccessful quest to obtain the squarate-based isoreticular analogue of CALF-20 (SquCALF-20), which was computationally predicted to outperform the parent MOF as a sorbent for postcombustion CO_2_ capture, led to the discovery of two new, nonporous coordination polymers, named **UdP-40** and **UdP-41**. These compounds share the same 2:2:1 Zn/trz/sqa stoichiometry expected for a CALF-20 analogue, however, their crystal structures significantly deviate from the one with **dmc** topology of CALF-20. The presence of tetradentate squarate ligands coordinated through only three of their four O atoms in **UdP-40** and **UdP-41** makes the desired **dmc** topology not accessible. No appreciable similarities are evident with the structure of recently reported squarate-based MAF-60 either, suggesting that the nature of the triazolate ligand also plays a key role in driving the formation of the framework when squarate is employed as the oxygenated ligand. Our synthetic screening suggests that SquCALF-20 is not experimentally accessible in conditions commonly used to synthesize CALF-20 and similar compounds. Based on this work, we suggest that, when new MOFs are predicted to outperform established benchmark materials, particular attention should be paid to their experimental synthetic accessibility.

## Supplementary Material





## Data Availability

The data underlying this study are openly available in Zenodo at 10.5281/zenodo.20540567.

## Notes

Additional PXRD patterns and structural figures (PDF)
CO_2_ adsorption isotherms in aif format (ZIP)
