# Supplementary material for: Squarate-Based Isoreticular Analogue of CALF-20: Fact or Fiction?
Source: Cryst Growth Des. 2026 Aug 6;26(16):6310–8. doi: 10.1021/acs.cgd.6c00193 (PMC13501916; doi:10.1021/acs.cgd.6c00193)
Supplement: Supplementary file 1 [file cg6c00193_si_001.pdf]

Squarate-based analogue of CALF-20: fact or fiction?

Francesco Della Croce,[1] Giulio Bresciani,[1,2,*] Marco Taddei[1,2,*]

[1]*Department of Chemistry and Industrial Chemistry, INSTM Research Unit, University of Pisa, Via G. Moruzzi 13, 56124, Pisa, Italy. Email: giulio.bresciani@unipi.it marco.taddei@unipi.it*

[b] *Centro per l'Integrazione della Strumentazione scientifica dell'Università di Pisa (C.I.S.U.P.), University of Pisa, 56124 Pisa, Italy*

SUPPORTING INFORMATION

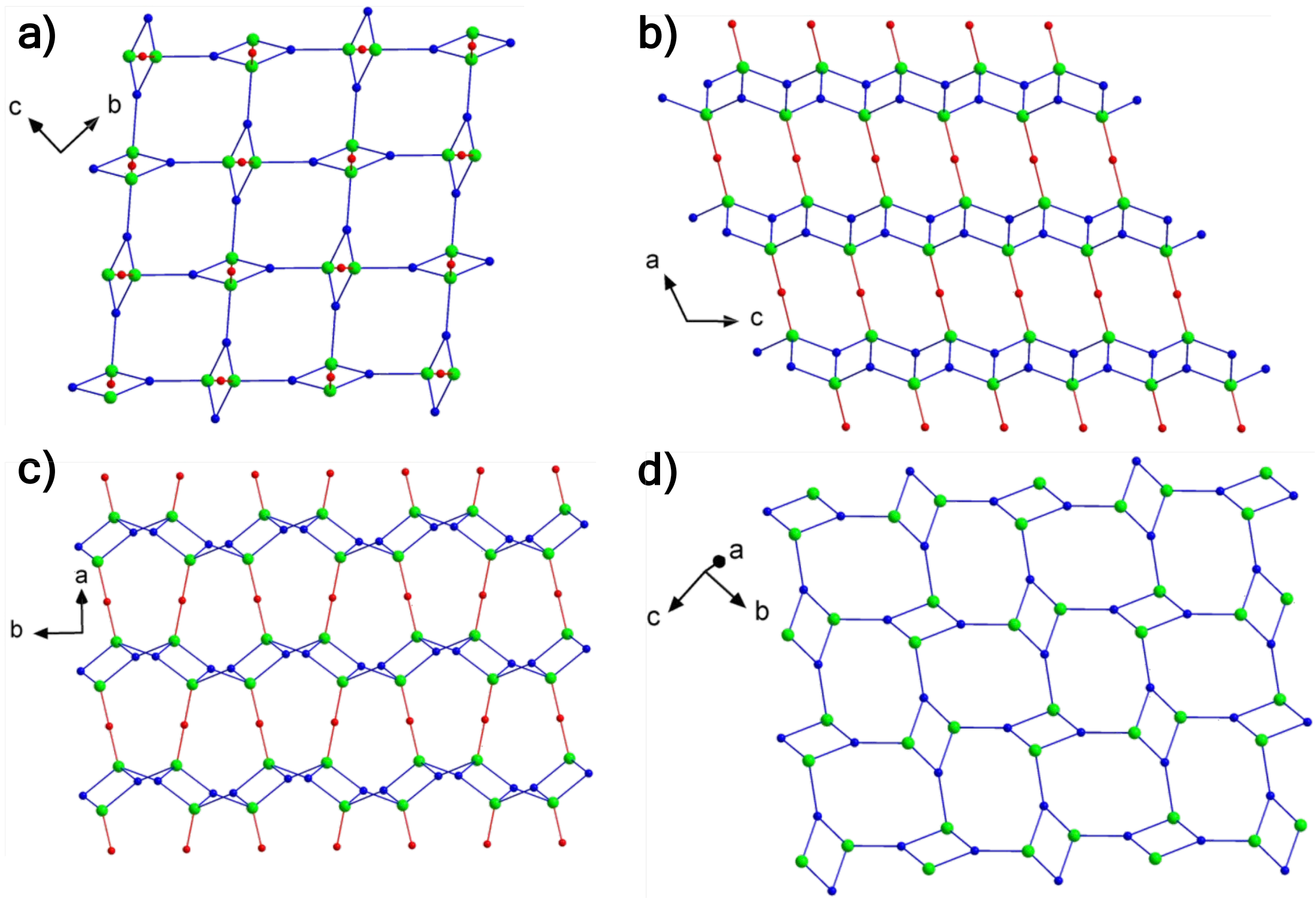

Figure S1. 3,4-connected **dmc** topology of CALF-20: along *a* axis (**a**), along *b* axis (**b**), along *c* axis (**c**) and along imidazole plane (**d**). Colour code: Zn, green; triazole centroid, blue; oxalate centroid, red.

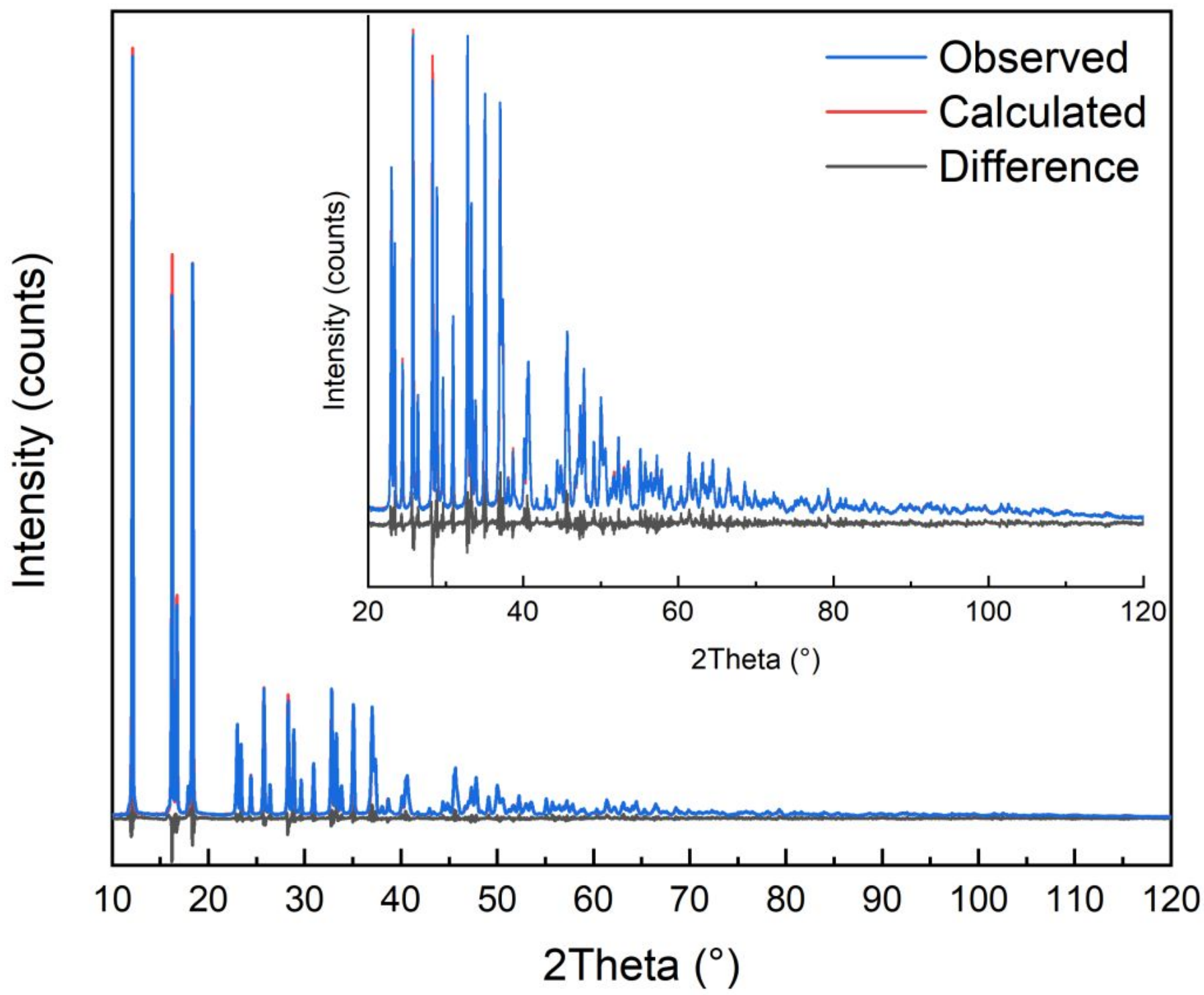

Figure S2. Rietveld plot for **UdP-40**.

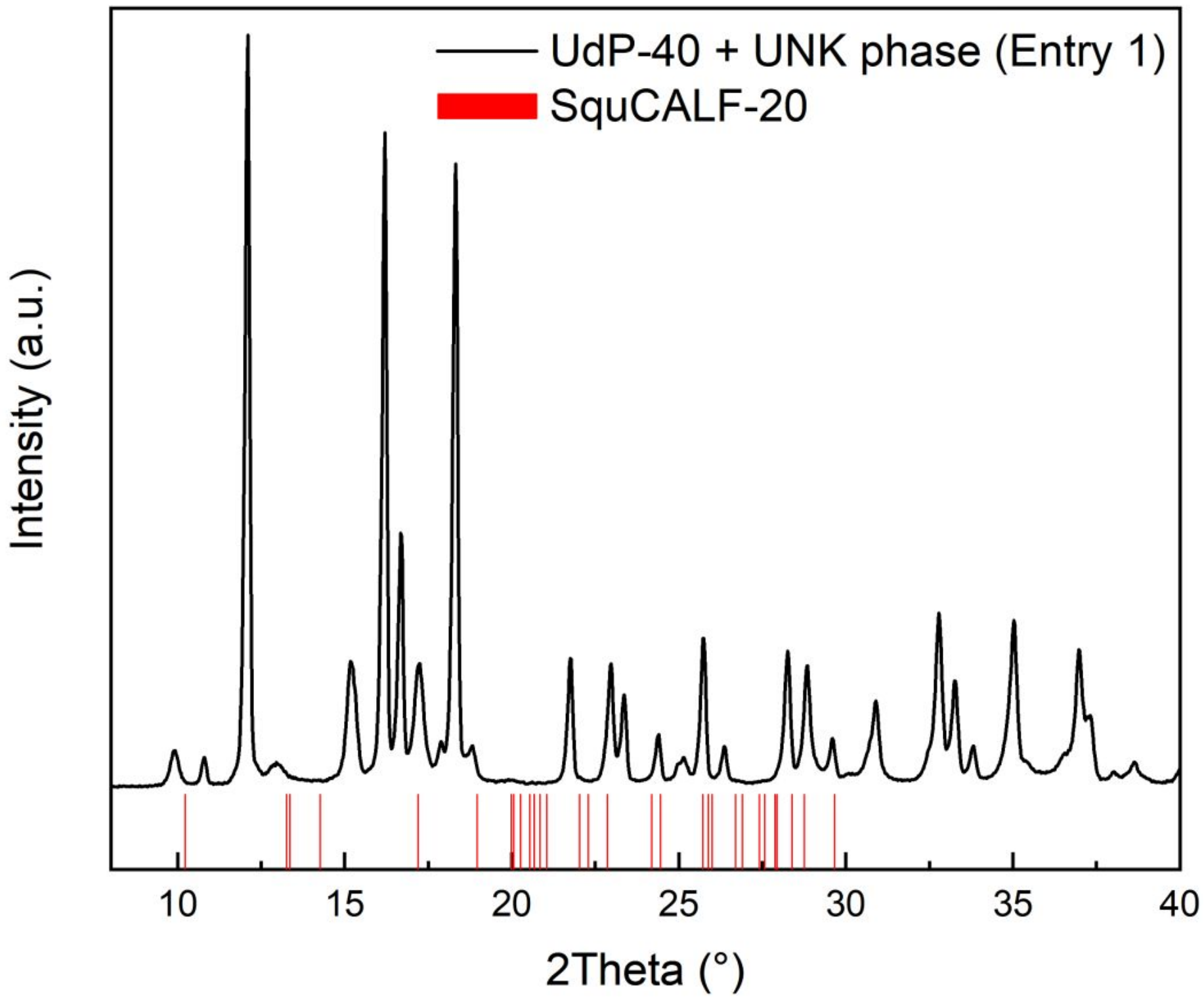

Figure S3. PXRD pattern of **UdP-40** mixed with an unknown phase (black line) compared with the calculated reflections (red lines) obtained from the cell parameters reported in Gopalsamy, K.*et al. ACS Appl. Eng. Mater*. **2024**, *1*, 96–103 (10.1021/acsaenm.3c00622).

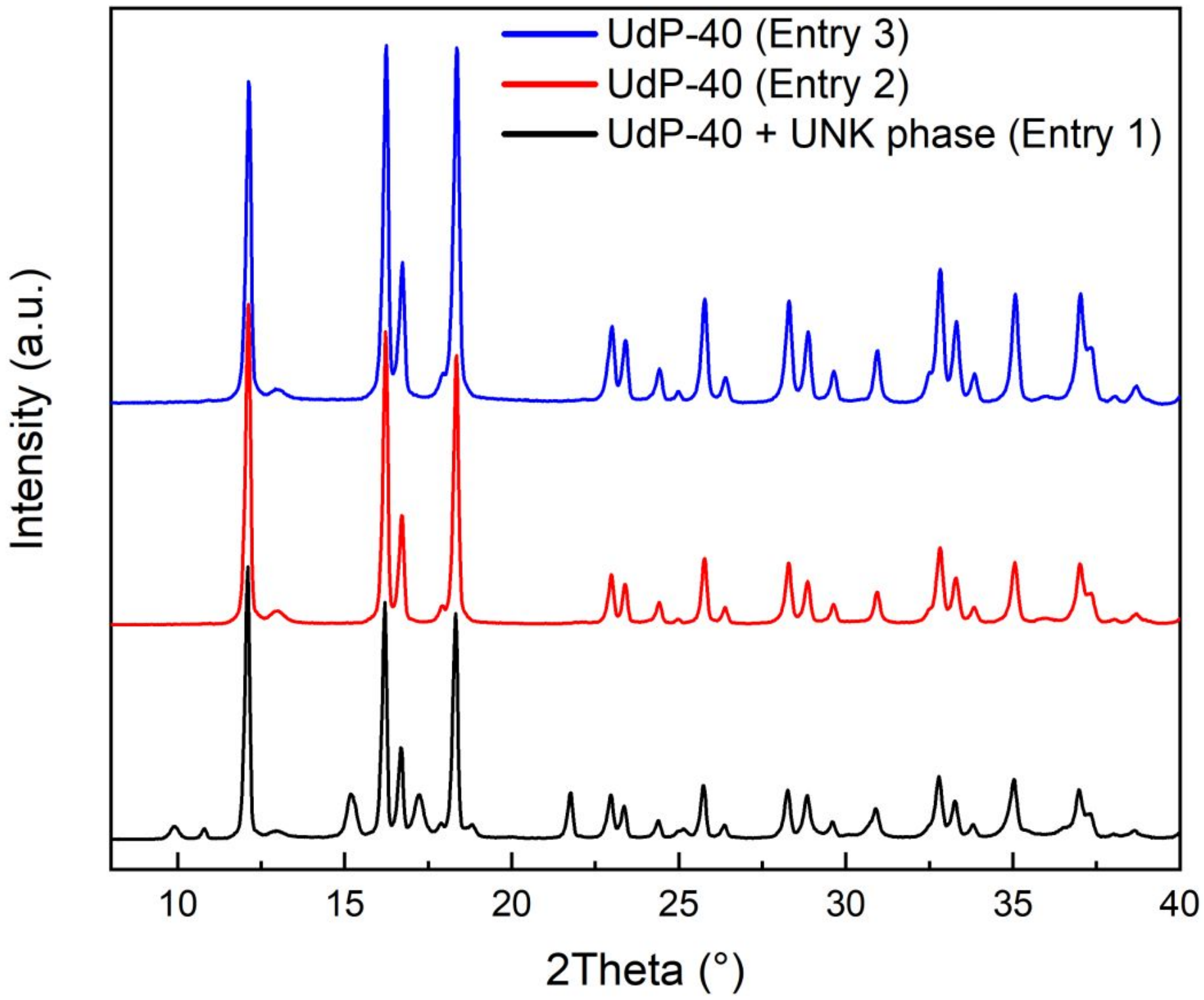

Figure S4. Comparison between PXRD patterns of materials prepared as reported in Entries 1, 2 and 3 in Table 3 in the main text.

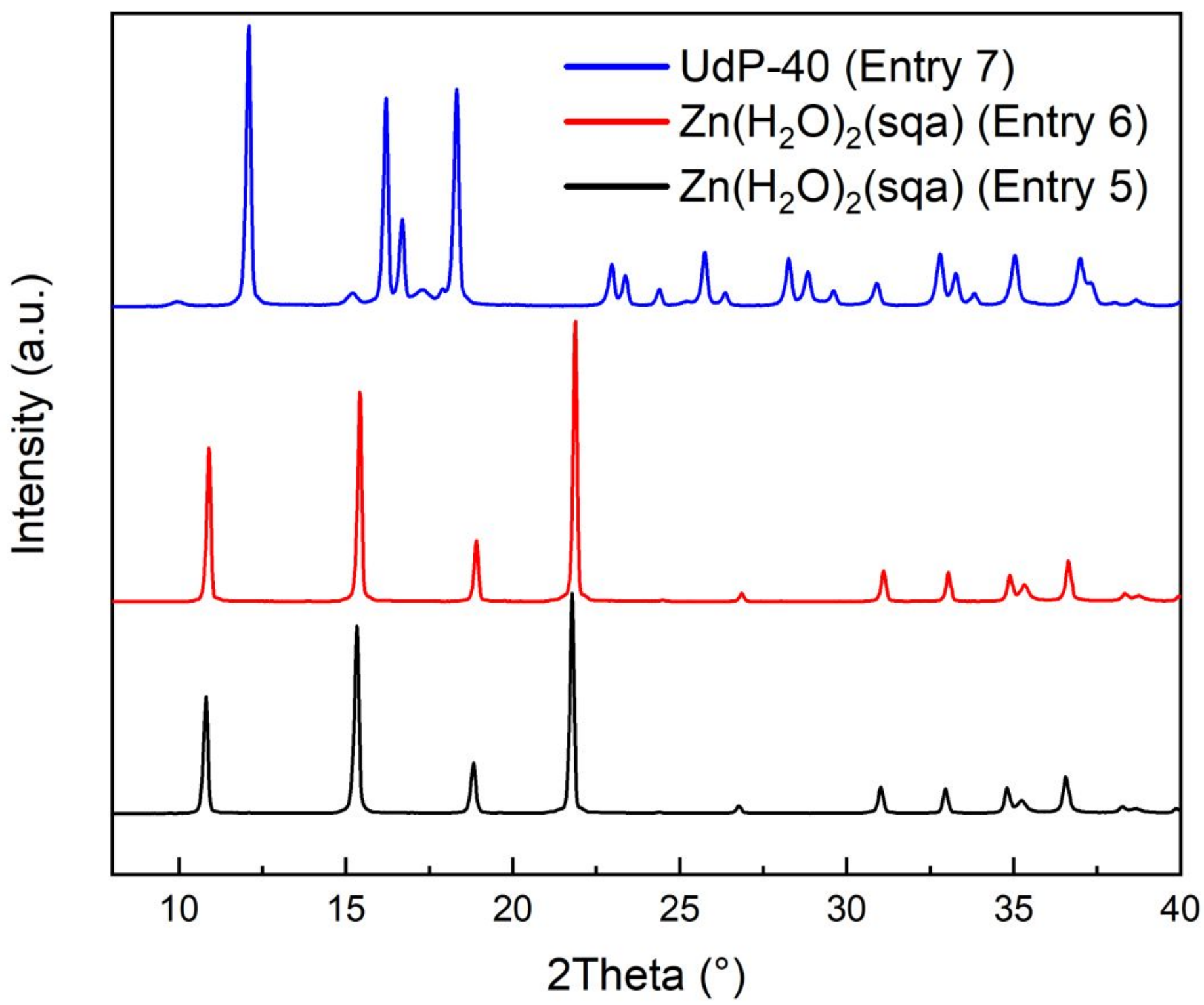

Figure S5. Comparison between PXRD patterns of materials prepared as reported in Entries 5, 6 and 7 in Table 3 in the main text. PXRD patterns of Entries 5 and 6 are consistent with a $Zn(H_2O)_2$(sqa) sodalite like topology.

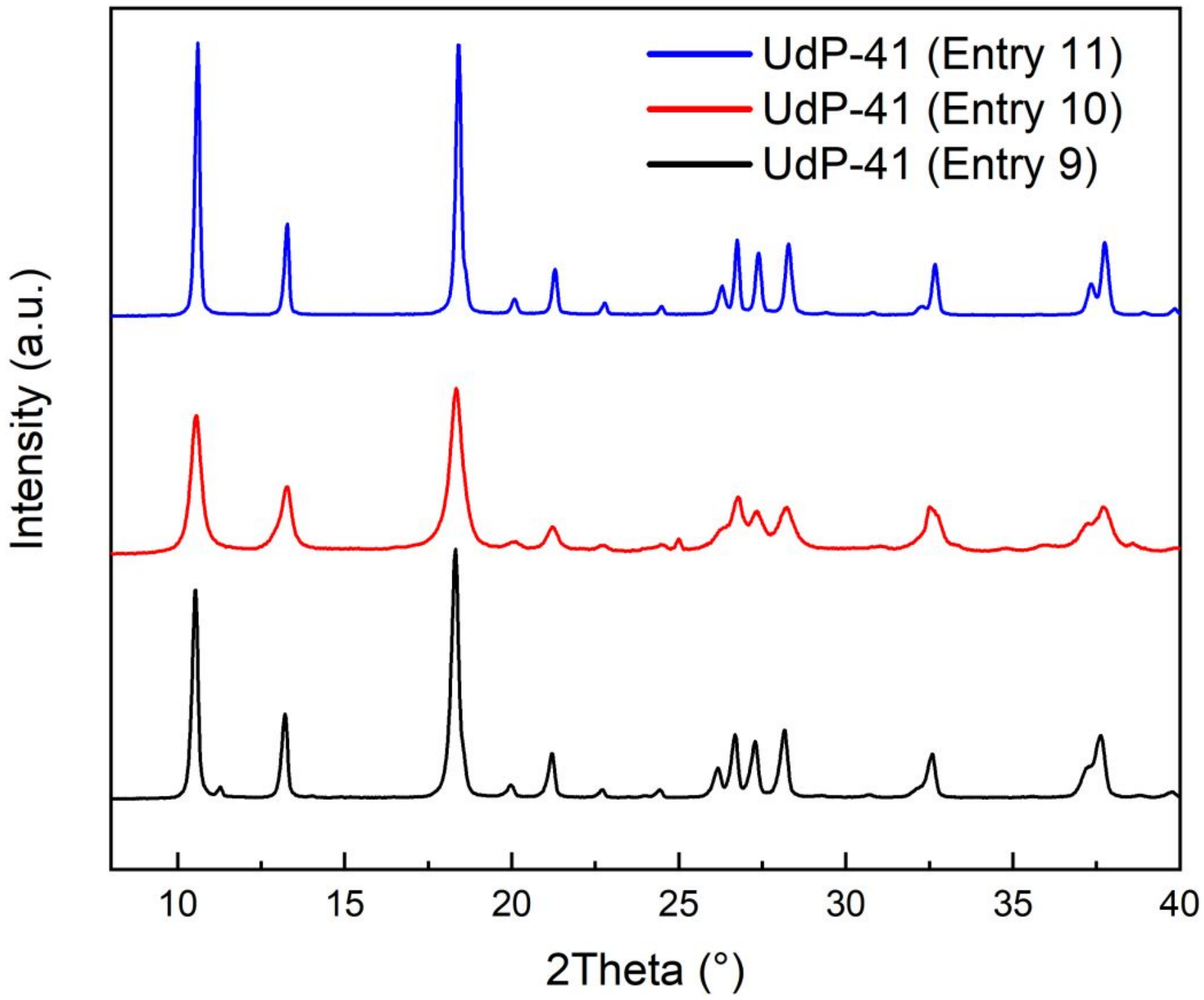

Figure S6. Comparison between PXRD patterns of materials prepared as reported in Entries 9, 10 and 11 in Table 3 in the main text.

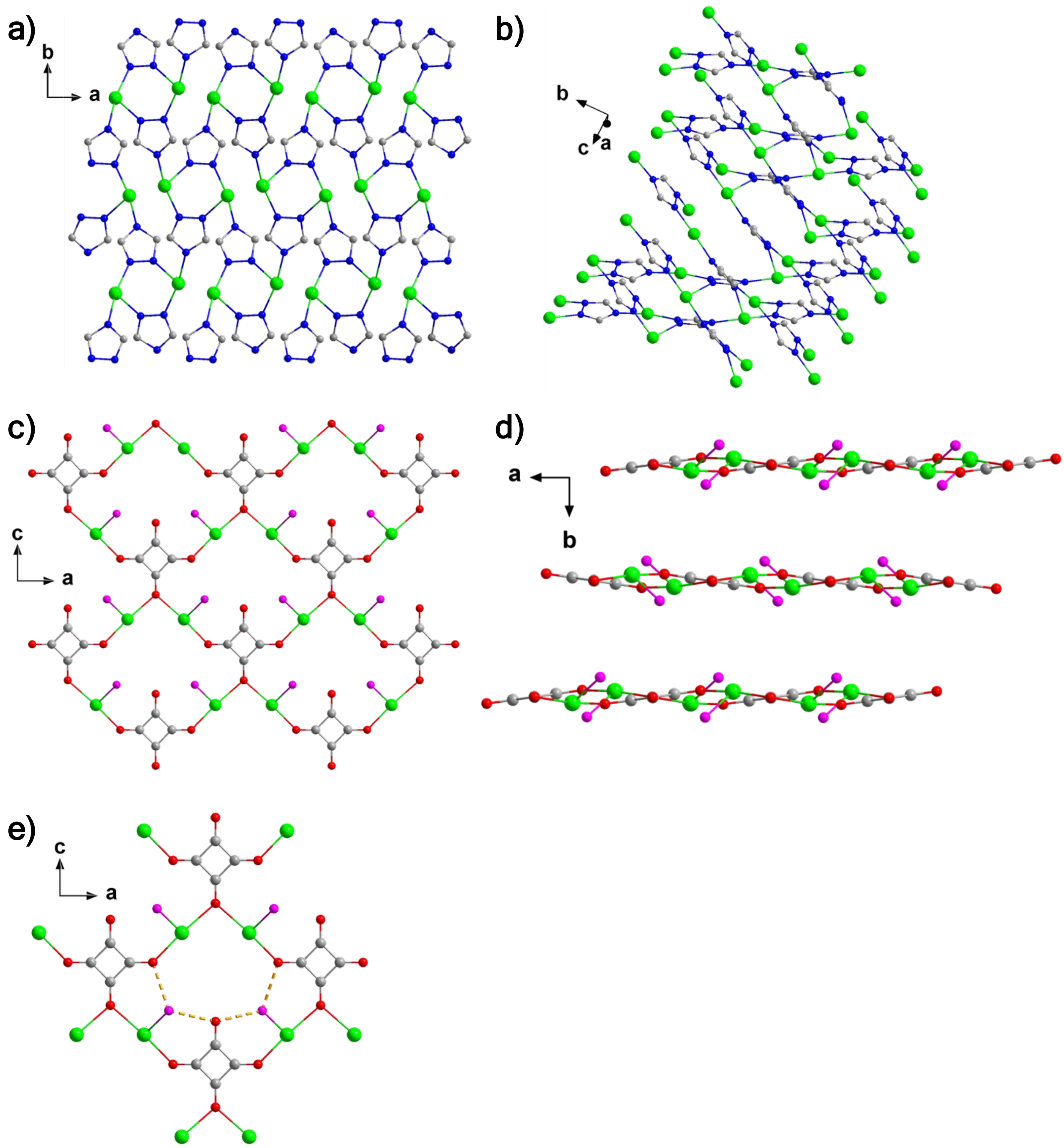

Figure S7. Sublattices in **UdP-40**: Zn-trz 3D sublattice **(a-b)**, Zn-sqa 2D sublattice **(c-d)**, and hydrogen bonds (in yellow) between coordinated water molecules and O9 and O10 in squarate ligands **(e)**. Colour code: Zn, green; N, blue; C, grey; O (sqa), red; O (water), pink.

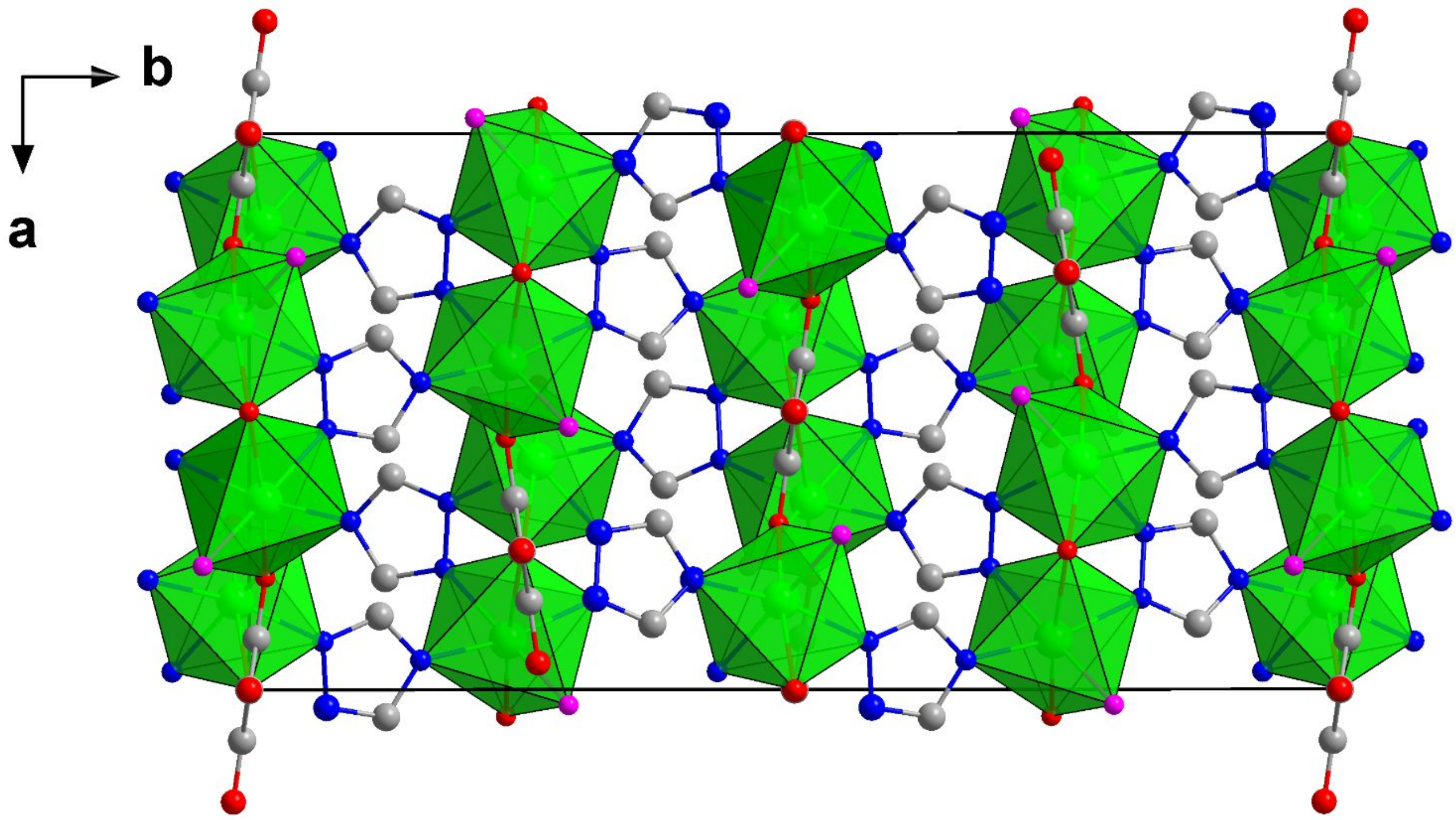

Figure S8. View of the crystal structure of **UdP-40** along the *c* axis. Colour code: Zn, green; N, blue; C, grey; O (squarate), red; O (water), purple.

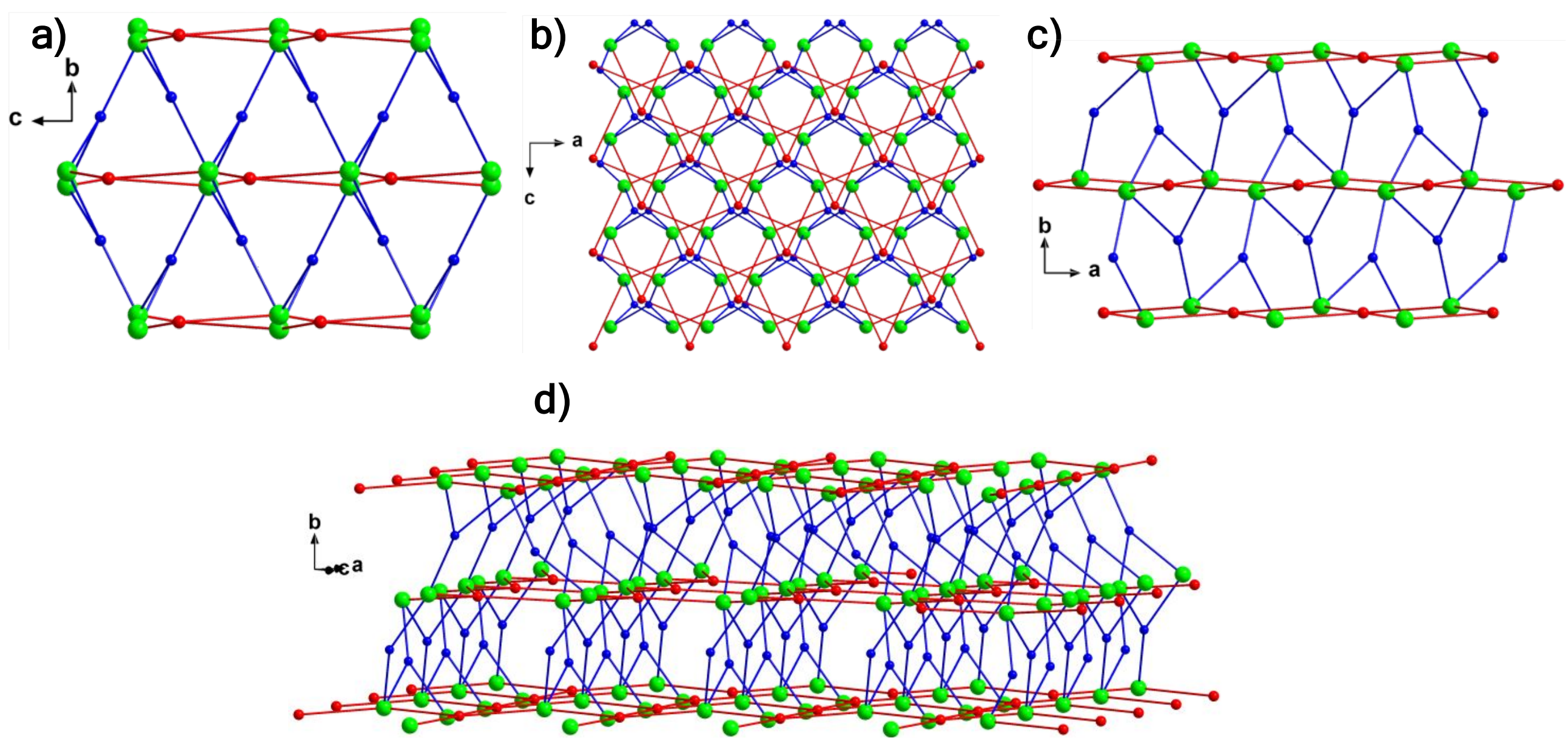

Figure S9. Topological reduction of **UdP-40**. Colour code: Zn, green; trz centroid, blue; sqa centroid, red.

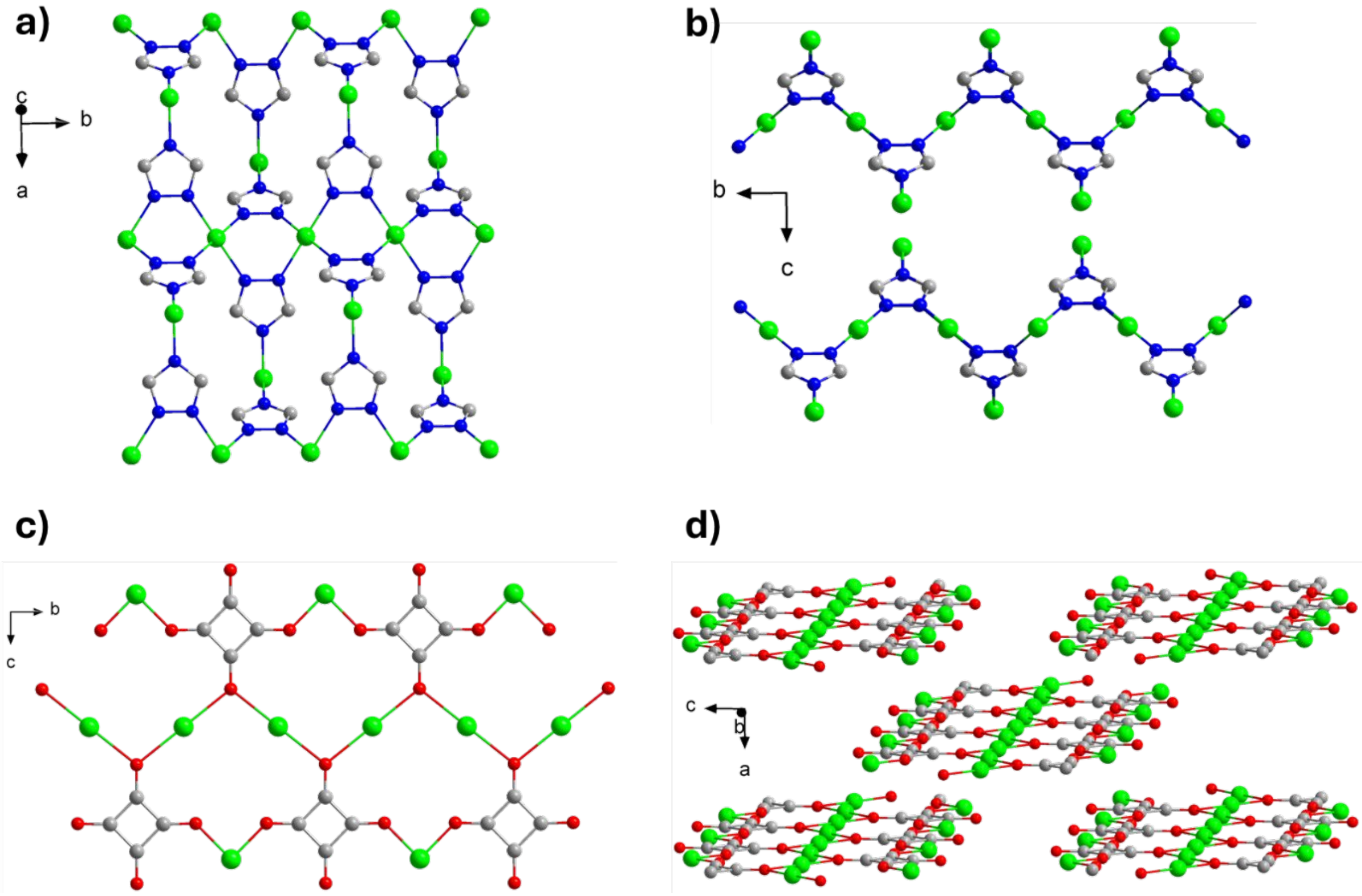

Figure S10. Sublattices in **UdP-41**: Zn-trz 2D sublattice **(a-b)** and Zn-sqa 1D sublattice **(c-d)**. Colour code: Zn, green; N, blue; C, grey; O, red. H atoms omitted for clarity.

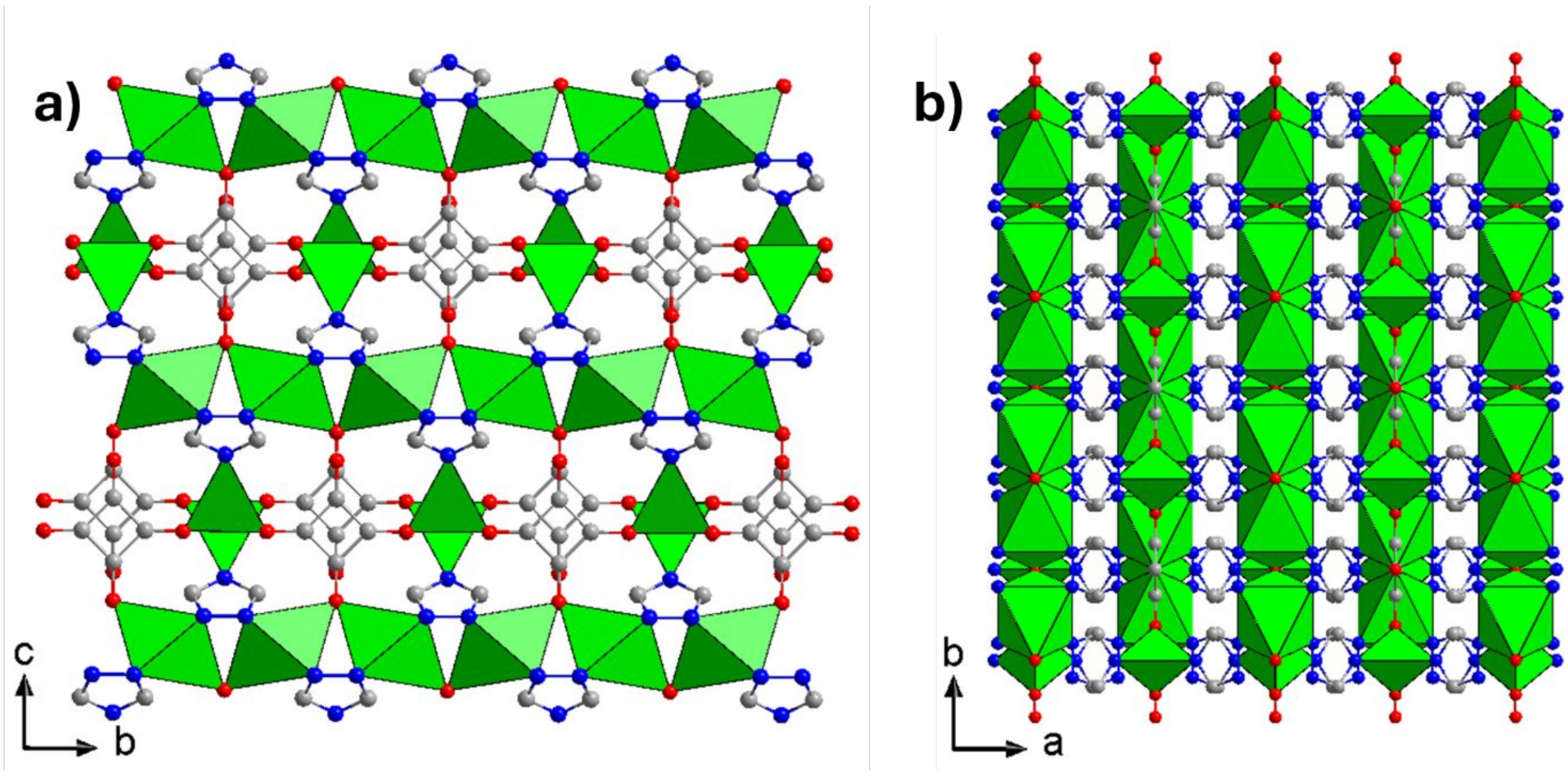

Figure S11. View of the structure of **UdP-41** along the *a* axis **(a)** and the *c* axis **(b)**. Colour code: Zn, green; trz centroid, blue; sqa centroid, red.

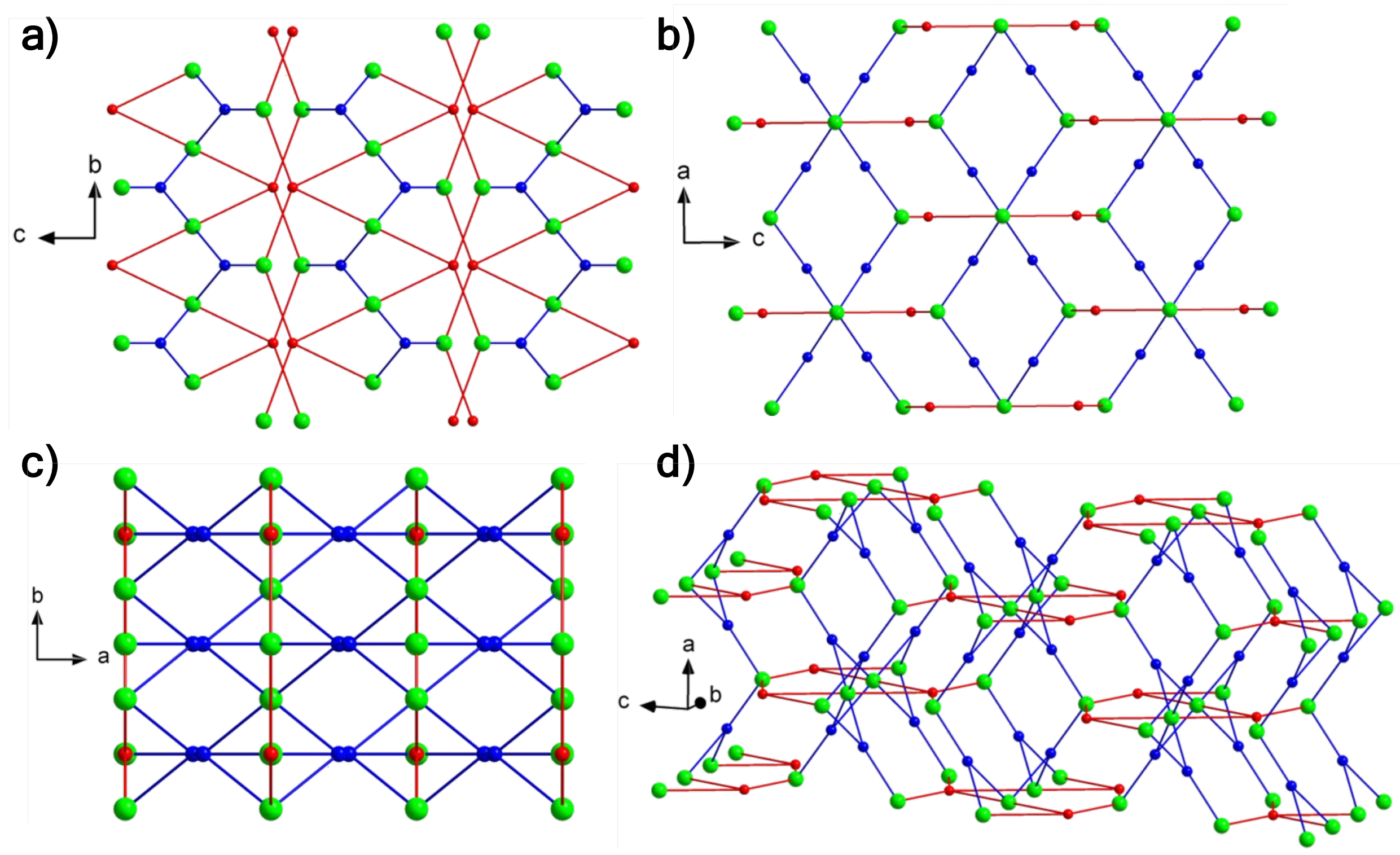

Figure S12. Topological reduction of **UdP-41**. Colour code: Zn, green; trz centroid, blue; sqa centroid, red.

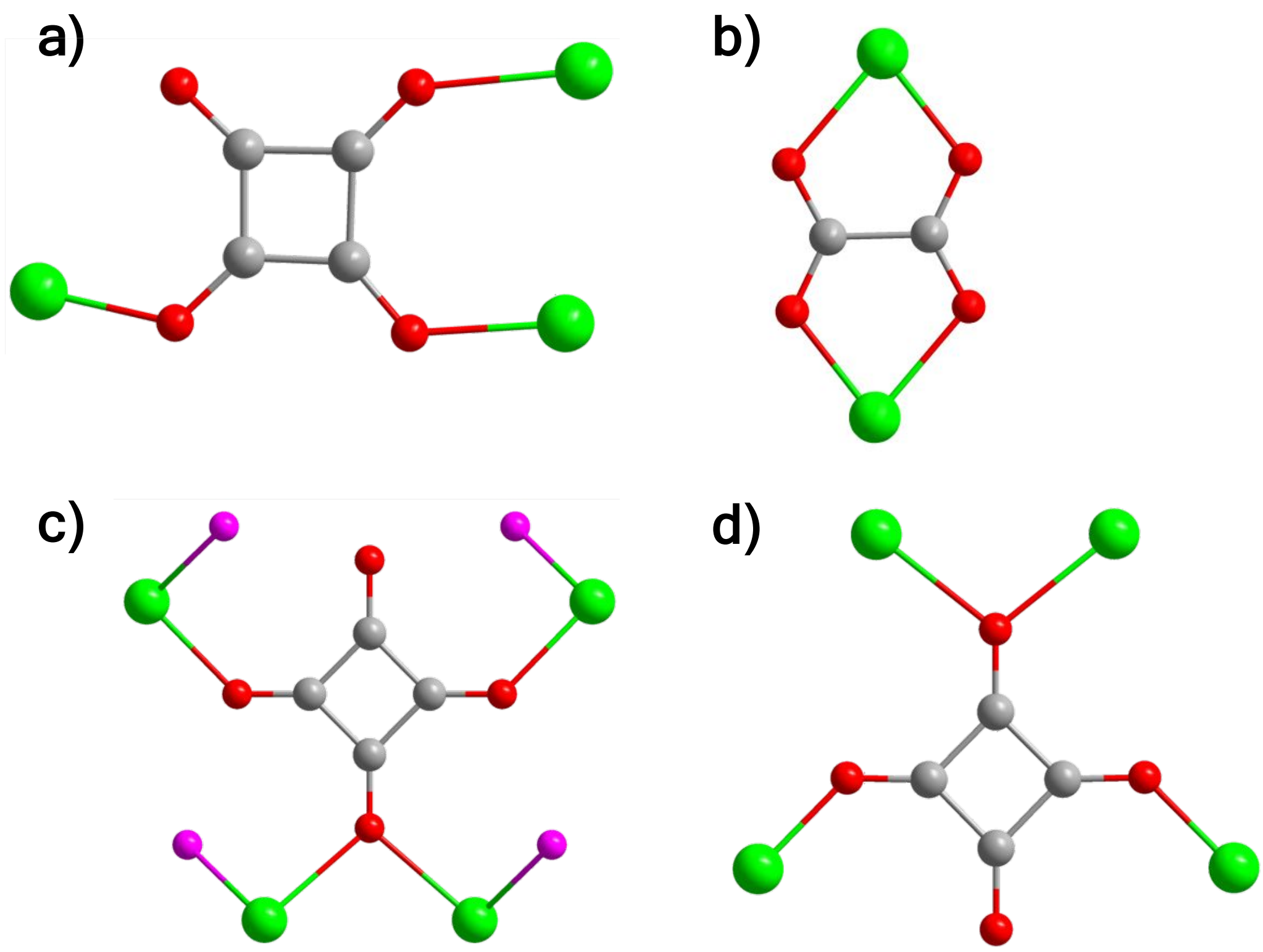

Figure S13. Coordination mode of the oxo ligand in MAF-60 **(a)**, CALF-20 **(b)**, **UdP-40 (c)** and **UdP-41 (d)**. Colour code: Zn, green; N, blue; C, grey; O (ligand), red; O (water), pink. Hydrogen atoms omitted for clarity.

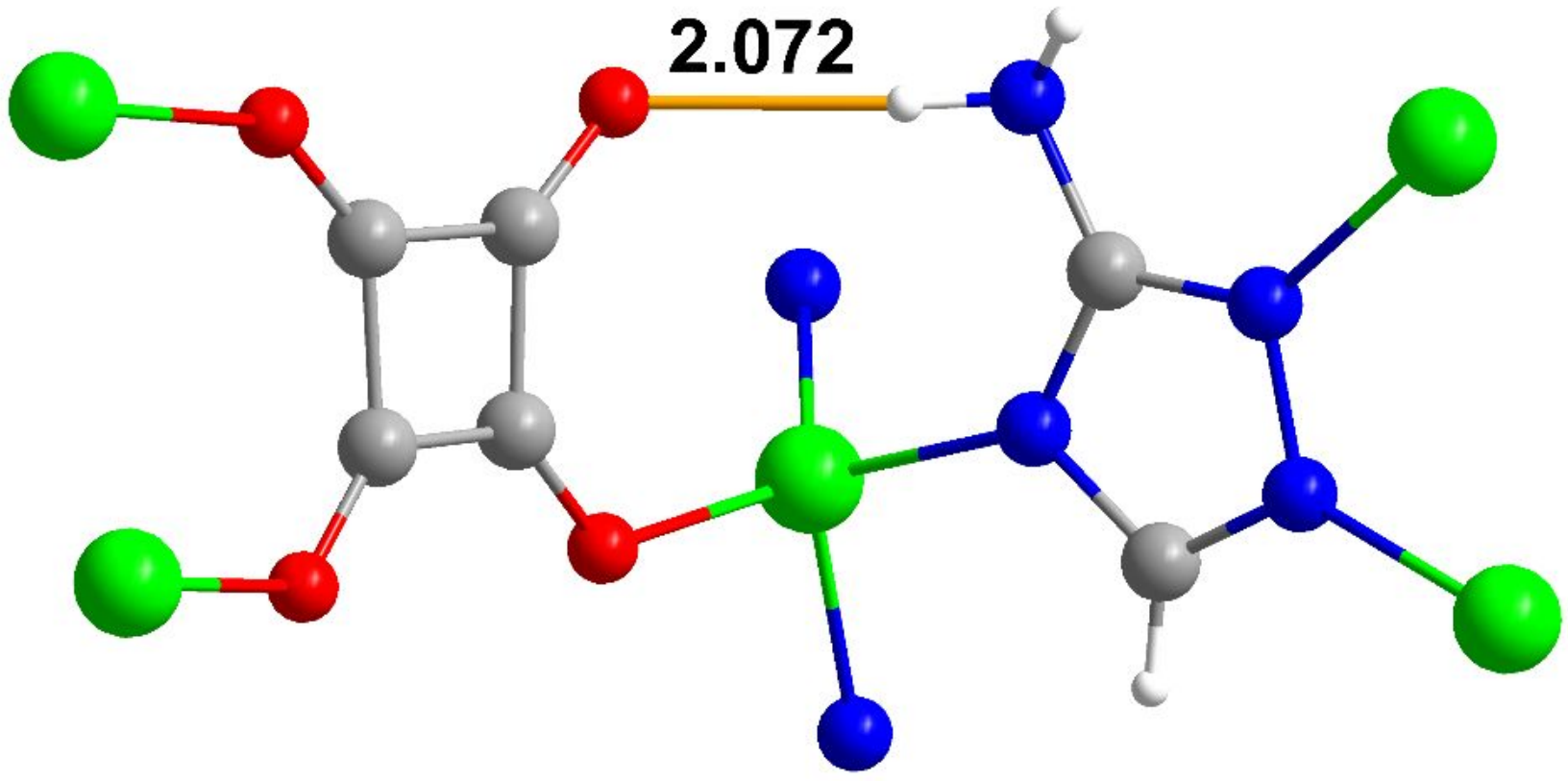

Figure S14. Hydrogen bond between amine functionality and uncoordinated oxygen of sqa ligand in **MAF-60**.

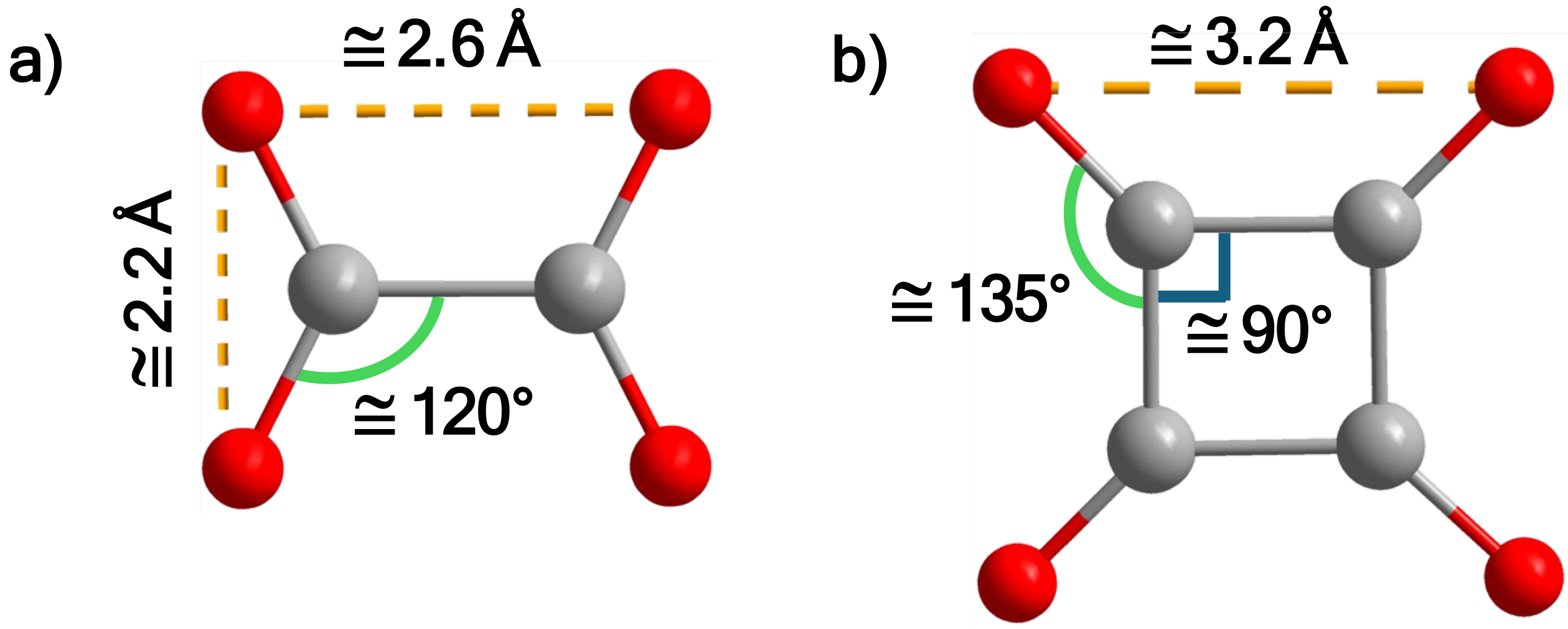

Figure S15. Relevant atomic distances and angles responsible for the different coordination and topological behaviour of oxalate **(a)** and sqa **(b)**.
